# Prevention of Pre-Eclampsia: Modern Strategies and the Role of Early Screening

**DOI:** 10.3390/jcm14092970

**Published:** 2025-04-25

**Authors:** Gulzhaina Alipova, Nurgul Ablakimova, Kymbat Tussupkaliyeva, Saule Bermagambetova, Sholpan Kosmuratova, Bibigul Karimsakova, Andrey Gaiday, Assel Gaiday, Andrii Dinets, Akylbek Tussupkaliyev

**Affiliations:** 1 Department of Obstetrics and Gynecology, West Kazakhstan Marat Ospanov Medical University, Aktobe 030012, Kazakhstan; 2 Department of Pharmacology, Clinical Pharmacology, West Kazakhstan Marat Ospanov Medical University, Aktobe 030012, Kazakhstan; 3 Department of Hospital Pharmacy, Regional Perinatal Center, Aktobe 030006, Kazakhstan; 4 Department of Epidemiology, West Kazakhstan Marat Ospanov Medical University, Aktobe 030012, Kazakhstan; 5 Department of Hygienic Disciplines and Occupational Diseases, West Kazakhstan Marat Ospanov Medical University, Aktobe 030012, Kazakhstan; 6 Department of Normal Physiology, West Kazakhstan Marat Ospanov Medical University, Aktobe 030012, Kazakhstan; 7 Department of General Medical Practice №1, West Kazakhstan Marat Ospanov Medical University, Aktobe 030012, Kazakhstan; 8 Department of Radiology, West Kazakhstan Marat Ospanov Medical University, Aktobe 030012, Kazakhstan; 9 Institute of Biology and Medicine, Taras Shevchenko National University of Kiyv, Kyiv 01033, Ukraine

**Keywords:** pre-eclampsia, early diagnosis, risk assessment, biomarkers, prenatal screening

## Abstract

**Background**: Pre-eclampsia (PE) remains a leading cause of maternal and perinatal morbidity and mortality worldwide. Early detection and risk stratification are critical for improving pregnancy outcomes. This review aims to summarize current advancements in PE screening, including clinical risk factors, biomarkers, imaging techniques, and predictive models. **Methods:** A comprehensive literature search was conducted using PubMed, Scopus, Web of Science, and Google Scholar to identify relevant studies on PE screening and prediction. Peer-reviewed original studies, systematic reviews, and meta-analyses published in English were included, while case reports and conference abstracts were excluded. **Results**: Traditional screening methods rely on maternal history and clinical risk factors, while emerging approaches incorporate biochemical markers and ultrasound parameters to enhance predictive accuracy. Machine learning models and artificial intelligence (AI)-driven algorithms are being explored for improved risk stratification. However, challenges such as data heterogeneity, lack of external validation, and integration into clinical practice remain. **Conclusions:** Advances in PE screening hold promise for early identification and targeted prevention strategies. Future research should focus on validating predictive models in diverse populations, integrating AI with traditional screening methods, and developing personalized approaches to reduce PE-associated complications.

## 1. Introduction

Pre-eclampsia (PE) is a hypertensive disorder of pregnancy characterized by high blood pressure and signs of organ dysfunction, most commonly involving the liver and kidneys, typically arising after 20 weeks of gestation [1]. It remains a major direct cause of maternal complications and death, and due to its underlying placental dysfunction, it is also linked to an increased risk of fetal growth restriction, stillbirth, preterm birth, and neonatal morbidity and mortality [2,3]. This pregnancy-related condition affects women globally, regardless of nationality, ethnicity, or age. Its prevalence varies widely, ranging from approximately 2% to 15% of pregnancies, with an average occurrence of around 4.6% [4,5].

PE is a major cause of adverse maternal and fetal outcomes worldwide, leading to increased healthcare costs. Studies from the United States of America (USA) and Ireland have highlighted the substantial economic burden of PE, with costs significantly higher for both mothers and infants compared to uncomplicated pregnancies [6,7]. For instance, research has shown that the cost of managing preeclamptic pregnancies averages USD 41,790, primarily driven by infant care, including extended neonatal intensive care unit (NICU) stays and preterm birth complications [6]. The additional costs stem from more intensive maternal care, higher rates of cesarean deliveries, and the need for specialized treatments [8]. Despite limited research, it is clear that PE imposes a significant financial strain on healthcare systems, emphasizing the need for adequate resource allocation to address its health and economic impacts.

Early detection and prevention are critical in managing PE, as timely intervention can significantly reduce maternal and fetal complications [9]. PE often develops without clear symptoms [10], making early screening essential for identifying women at risk. Early detection allows for risk stratification, enabling healthcare providers to implement appropriate interventions, such as pharmacological treatments or lifestyle modifications, to prevent or mitigate the condition. This review aims to explore modern strategies for preventing PE, emphasizing the role of early screening through clinical tools, biomarkers, and ultrasound parameters. By examining the latest research, this article highlights how early detection can improve pregnancy outcomes and guide targeted prevention, ultimately reducing the burden of PE on maternal and fetal health.

## 2. Methodology

A comprehensive literature search was conducted using PubMed, Scopus, Web of Science, and Google Scholar to identify relevant studies on PE screening and prediction. Eligible articles included peer-reviewed original studies, systematic reviews, and meta-analyses published in English, focusing on risk assessment, biomarkers, imaging techniques, and predictive models. Case reports, conference abstracts, and studies lacking relevant data on early detection and prevention strategies were excluded. The findings were synthesized into a qualitative summary, highlighting current advancements, challenges, and future prospects in pre-eclampsia screening and prevention.

## 3. Pathophysiology of Pre-Eclampsia

**Figure 1:**
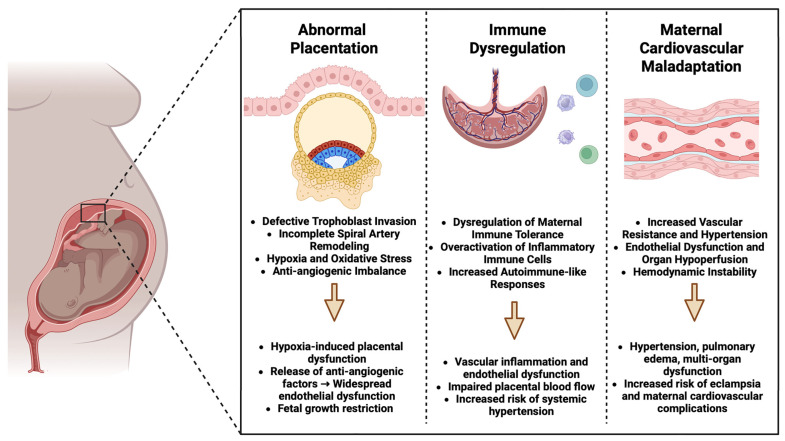
The pathogenesis of pre-eclampsia: placental, immune, and cardiovascular interactions.

PE is a complex hypertensive disorder of pregnancy characterized by endothelial dysfunction, systemic inflammation, and multi-organ involvement [11]. PE can be described as a two-stage process. The first stage, occurring in early pregnancy (first trimester), involves abnormal placentation. This is followed by a symptomatic phase after 20 weeks of gestation, characterized by maternal hypertension and multiorgan dysfunction [12]. Although the exact pathophysiology has not been fully elucidated, existing evidence indicates that it results from a complex interplay of factors, including impaired placental development, immune system disturbances, and maternal cardiovascular maladaptation (Figure 1).

During normal pregnancy, extravillous trophoblasts invade the maternal spiral arteries, transforming them into low-resistance vessels to ensure adequate placental perfusion [13]. However, in PE, this process is impaired, leading to incomplete spiral artery remodeling [14]. As a result, these blood vessels stay constricted with high resistance, leading to placental hypoxia and increased oxidative stress, which, in turn, play a role in endothelial dysfunction and systemic inflammation [15,16]. Placental hypoxia leads to an elevated release of antiangiogenic factors, including soluble fms-like tyrosine kinase-1 (sFlt-1) and soluble endoglin (sEng), which interfere with the equilibrium of proangiogenic factors such as vascular endothelial growth factor (VEGF) and placental growth factor (PlGF) [17]. This imbalance ultimately leads to endothelial dysfunction, reduced nitric oxide availability, increased vascular permeability, and hypertension [18].

Endothelial injury is a hallmark of PE, contributing to widespread vascular dysfunction [19]. In addition, increased circulating inflammatory cytokines, such as tumor necrosis factor-alpha (TNF-α) and interleukin-6 (IL-6), further exacerbate endothelial damage [20]. This proinflammatory state, coupled with oxidative stress, promotes vasoconstriction, platelet aggregation, and microvascular injury, which collectively lead to the clinical manifestations of PE [21]. Furthermore, PE is associated with increased systemic vascular resistance and reduced cardiac output [22]. The kidneys exhibit glomerular endotheliosis, characterized by the swelling of endothelial cells, which results in proteinuria and decreased renal function [23,24]. Additionally, impaired natriuresis and activation of the renin–angiotensin–aldosterone system (RAAS) further contribute to hypertension and fluid retention [25,26].

Another key factor in the development of PE is altered maternal immune tolerance to fetal antigens. Specifically, aberrant interactions between maternal natural killer (NK) cells and trophoblastic cells at the boundary between maternal and fetal tissues may lead to insufficient trophoblast invasion and placental ischemia [27]. Moreover, an imbalance between regulatory T cells (Tregs) and proinflammatory immune cells exacerbates systemic inflammation, further contributing to disease progression [28,29]. Along with immune system disturbances, genetic factors contribute to the development of PE, as the condition is more frequently observed in women with a family history of the disease [30,31,32]. Additionally, epigenetic changes, including DNA methylation and microRNA activity, can impact placental gene expression, leading to disrupted placental formation and altered maternal vascular function [33,34].

Understanding these interconnected mechanisms is crucial for developing early screening strategies and targeted prevention approaches to mitigate the risks associated with this condition.

## 4. Risk Factors for Pre-Eclampsia

PE develops due to a combination of genetic, physiological, and environmental influences. Multiple risk factors contribute to the development of this condition, encompassing maternal characteristics, genetic predisposition, and lifestyle influences. Specific maternal factors that elevate the risk of PE include advanced maternal age (over 35 years) [4,35], elevated body mass index (BMI) or obesity [36,37], pre-existing conditions such as hypertension, diabetes mellitus, or renal disease [38], history of PE in prior pregnancies [39], multifetal pregnancies including twins or triplets [40,41], autoimmune disorders like lupus or antiphospholipid syndrome [42,43], and the use of assisted reproductive technologies such as in vitro fertilization [44]. The role of obesity-related conditions in the development of PE is evident, as it is more common in women with metabolic disorders like polycystic ovary syndrome or insulin resistance [45]. Genetic factors also play a significant role in PE, as a family history of the condition in a mother or sister increases the risk. Khan et al. found that 36.67% of women had a family history of PE [46], while a 2021 study using Taiwan’s National Health Insurance Database reported a lower prevalence of 12.17% [31], with these women also facing a heightened risk of hypertension. Additionally, specific genetic variants related to immune function, angiogenesis, and vascular regulation have been linked to its development, while epigenetic factors, including DNA methylation and microRNA expression, may further influence the likelihood of the disorder [47,48]. Environmental and lifestyle influences contribute significantly to PE risk, including poor diet lacking essential nutrients such as calcium and antioxidants [49,50], a physically inactive lifestyle, elevated chronic stress levels, various psychosocial influences, and exposure to environmental pollutants such as air contaminants and heavy metals, along with habits like smoking and excessive alcohol intake [51,52]. A 2016 prospective cohort study in Australia reported that women who took a multivitamin and mineral supplement during the first trimester of pregnancy had a 67% reduced risk of developing PE [53]. Recognizing and addressing these risk factors is essential for developing effective prevention strategies and improving maternal and fetal outcomes.

## 5. Screening Methods for Early Detection of Pre-Eclampsia

### 5.1. Clinical Screening Tools

Early detection through effective screening methods is essential for timely intervention and the management of PE. Various screening strategies include clinical tools, biochemical markers, ultrasound parameters, and combination models for risk prediction. Clinical assessment remains a cornerstone of PE screening. Essential approaches involve evaluating maternal history and recognizing risk factors, including chronic hypertension, obesity, diabetes, and a prior history of PE.

A thorough systematic review and meta-analysis of 92 studies, covering 25,356,688 pregnancies, investigated the association between clinical risk factors present before 16 weeks of gestation and the probability of developing PE. The most significant risk factors included a previous history of PE (RR 8.4, 95% CI 7.1–9.9) and chronic hypertension (RR 5.1, 95% CI 4.0–6.5). Other contributing factors were nulliparity (RR 2.1, 95% CI 1.9–2.4), maternal age over 35 years (RR 1.2, 95% CI 1.1–1.3), chronic kidney disease (RR 1.8, 95% CI 1.5–2.1), conception through assisted reproductive technology (RR 1.8, 95% CI 1.6–2.1), a pre-pregnancy BMI above 30 kg/m^2^ (RR 2.8, 95% CI 2.6–3.1), and pregestational diabetes mellitus (RR 3.7, 95% CI 3.1–4.3) [54].

Various professional organizations, such as the National Institute for Health and Care Excellence (NICE) and the American College of Obstetricians and Gynecologists (ACOG) (Table 1), advocate for PE screening based on maternal risk factors. NICE advises that women with at least one high-risk factor—such as chronic hypertension, renal disease, diabetes, or autoimmune conditions—or multiple moderate-risk factors, including first-time pregnancy, maternal age of 40 or older, BMI of 35 or higher, a family history of PE, or an interpregnancy interval exceeding 10 years, should be classified as high-risk. These women are recommended to take 75–150 mg of aspirin daily from the 12th week of gestation until delivery [55]. Similarly, ACOG suggests low-dose aspirin for women with a prior pregnancy complicated by early-onset PE or multiple pregnancies affected by PE [56]. The United States Preventive Services Task Force (USPSTF) offers a more comprehensive guideline, which is now supported by ACOG, the Society for Maternal-Fetal Medicine, and the American Diabetes Association, recommending a daily dose of 81 mg of aspirin from 12 to 28 weeks for women with either one high-risk factor or multiple moderate-risk factors [57,58,59].

However, these screening methods have shown limited effectiveness. The NICE method identifies 41% of preterm PE cases and 34% of term PE cases, with a false-positive rate of 10%. In contrast, ACOG’s 2013 guideline detects just 5% of preterm and 2% of term PE cases, with a significantly lower false-positive rate of 0.2% [61]. The USPSTF model improves detection rates to 90% and 89% but increases the false-positive rate to 64% [62]. Most recommendations are based on retrospective epidemiologic studies, and current approaches do not differentiate risk by PE severity.

### 5.2. Biochemical Markers

Mammalian placentation relies on extensive angiogenesis to establish a functional vascular network that ensures adequate oxygen and nutrient supply to the fetus. This process is carefully controlled by the interplay between proangiogenic factors, including VEGF and PlGF, and antiangiogenic factors like sFlt-1, which is synthesized by the developing placenta. An imbalance in this system, especially an overproduction of antiangiogenic factors, contributes to widespread endothelial dysfunction, a key feature of PE. Notably, while maternal circulation exhibits abnormal levels of these factors, fetal concentrations remain unaffected and fetuses of mothers with PE do not develop the same clinical symptoms, such as hypertension and proteinuria [63].

VEGF and PlGF are essential angiogenic factors, while sFlt-1 and sEng have anti-angiogenic effects, influencing the pathogenesis of PE [64]. The serum levels of these factors vary between women with PE and those with normotensive pregnancies, highlighting their diagnostic and prognostic potential [64,65,66]. VEGF-A, particularly VEGF-A165, promotes vascular permeability, endothelial proliferation, and trophoblast regulation via VEGFRs, while PlGF, primarily binding to sFlt-1, contributes to non-branching angiogenesis in the second trimester [67].

sFlt-1, a soluble variant of VEGFR-1, inhibits VEGF and PlGF by binding them and preventing their signaling, with increased levels observed in PE [68]. The exact triggers for elevated sFlt-1 production by the placenta remain unclear. The most probable cause is placental ischemia [69]. In vitro studies indicate that trophoblasts have a distinctive ability to increase sFlt-1 production when oxygen levels are low [70]. This aligns with the observation that individuals who develop PE exhibit increased expressions of hypoxia-inducible transcription factors (HIFs) in their placentas [71]. However, it is uncertain whether elevated sFlt-1 secretion directly contributes to the early placental abnormalities seen in PE or if it occurs as a secondary response to ischemia triggered by another factor. Additionally, genetic predisposition and placental size, such as in multiple gestations, may also influence excessive sFlt-1 production. The regulation of sFlt-1 remains incompletely understood; however, recent research indicates that epidermal growth factor receptor and mitochondrial signaling pathways contribute to its placental release and the progression of PE [72].

Thadhani et al. [73] suggested that measuring serum levels of sFlt-1 and PlGF in the first trimester could aid in identifying women at high risk for PE. Subsequent studies confirmed that plasma sFlt-1 concentrations start to rise approximately 6–10 weeks before the onset of PE, reaching their highest levels 2–5 weeks prior to diagnosis. This increase was observed in both early-onset (EOPE) and late-onset PE (LOPE), with EOPE showing an earlier elevation. These findings indicate that the most suitable timeframe for diagnostic testing is between 28 and 32 weeks for EOPE and between 30 and 34 weeks for LOPE [74].

Buhimschi et al. demonstrated that the urinary sFlt-1-to-PlGF (uFP) ratio effectively differentiates severe PE from normotensive pregnancies and other hypertensive conditions [75]. Likewise, Hirashima et al. established reference values for serum sFlt-1, PlGF, and their ratio, facilitating risk assessment across different stages of pregnancy [76]. Their findings indicated that serum sFlt-1 levels increase significantly between 35 and 39 weeks, whereas PlGF reaches its peak between 26 and 30 weeks before declining, highlighting the need for gestation-specific cutoff values [76].

Ohkuchi et al. confirmed that sFlt-1/PlGF ratios at ~28 weeks predicted severe PE in 83% of cases [77]. Levine et al. reported that the ratio increases 2–3 months before PE onset, outperforming single biomarkers in predictive accuracy [78]. Stepan et al. demonstrated higher sFlt-1 and lower PlGF in complicated pregnancies, especially EOPE [79].

De Vivo et al. noted that while the sFlt-1/PlGF ratio increases during pregnancy in both healthy individuals and those with PE, the elevation is significantly greater in PE cases [80]. Romero et al. highlighted early shifts in pro- and anti-angiogenic factors in pregnancies complicated by fetal growth restriction (FGR) and PE [81].

Given the limitations of ELISA-based studies, automated electrochemiluminescence assays were introduced for rapid sFlt-1 and PlGF measurement. Ohkuchi et al. demonstrated that the sFlt-1/PlGF ratio, with an 85 cutoff, provided strong diagnostic accuracy for EOPE and LOPE within 18 min [77]. Verlohren et al. confirmed the reliability of this approach, demonstrating a sensitivity of 82% and a specificity of 95%, with even greater accuracy observed for EOPE [82]. Other automated platforms, such as Elecsys (Roche) and Beckman Coulter assays, further confirmed the ratio’s diagnostic value [83].

Chaiworapongsa et al. showed that angiogenic factor measurements could predict preterm delivery in severe PE cases [84]. Rana et al. found that an sFlt-1/PlGF ratio > 85 predicted adverse outcomes within two weeks, surpassing standard laboratory tests [85]. Moore et al. reinforced these findings, showing that integrating the ratio into clinical models improved risk stratification for PE complications [86]. In a multicenter study, Verlohren et al. confirmed these results using the Elecsys system [87].

A recent systematic review and meta-analysis encompassing eight studies found that pregnant women with FGR and PE tend to have a higher sFlt-1/PlGF ratio. The findings suggest that an sFlt-1/PlGF ratio above 33 is a strong indicator of FGR, making it a useful marker for identifying affected pregnancies. Additionally, a ratio of 85 or higher was associated with an increased likelihood of both FGR and PE, highlighting its potential role in predicting more severe pregnancy complications [88]. Another systematic review specifically examined the role of the sFlt-1/PlGF ratio in twin pregnancies. A total of 11 studies were analyzed, revealing that pregnancies with complicated PE or other adverse perinatal outcomes consistently showed elevated sFlt-1/PlGF ratios compared to uncomplicated pregnancies [89]. Limited data are available regarding the ratio’s variations in healthy twin pregnancies and differences based on chorionicity. However, these findings further reinforce the clinical value of the sFlt-1/PlGF ratio in identifying pregnancies at risk for placental dysfunction-related complications.

Uric acid, endoglin, and angiogenic factors such as VEGF and sVEGFR1 have also been studied, but they are considered secondary markers due to their lack of specificity or weaker association with PE [90,91]. Additionally, altered alpha-fetoprotein (AFP) levels and decreased plasma nitric oxide metabolites have been observed in some studies, but these are not widely adopted as primary predictive markers [92,93]. The most suitable biomarkers for PE often depend on the specific clinical context, but sFlt-1 and PlGF are currently considered the most promising and widely studied. The sFlt-1/PlGF ratio is especially valuable, with elevated sFlt-1 and low PlGF levels being strongly associated with the development of PE. This ratio has shown high predictive accuracy, making it a leading biomarker for assessing endothelial dysfunction and predicting PE risk.

The sFlt-1/PlGF ratio is especially valuable, with elevated sFlt-1 and low PlGF levels being strongly associated with the development of PE. This ratio has shown high predictive accuracy, making it a leading biomarker for assessing endothelial dysfunction and predicting PE risk. Importantly, recent health–economic evaluations have demonstrated that using this biomarker in clinical practice is not only diagnostically beneficial but also cost-effective. Table 2 summarizes key findings from cost-effectiveness studies conducted in various countries.

### 5.3. Ultrasound Parameters

Ultrasound has emerged as a valuable tool for PE risk assessment. Several sonographic parameters have been investigated for their role in predicting PE, particularly in high-risk populations. Uterine artery Doppler velocimetry is one of the most studied ultrasound modalities for PE prediction [105]. Abnormal Doppler findings, such as increased pulsatility index (PI) and the presence of diastolic notches, indicate impaired trophoblastic invasion and reduced placental perfusion [106]. Elevated PI in the first or second trimester has been associated with a higher risk of EOPE. Recent updated meta-analysis showed that the uterine artery PI measured by Doppler ultrasound has moderate sensitivity (0.59) and high specificity (0.88) for predicting PE [107]. Subgroup analysis indicated that the timing of ultrasound scans before 20 weeks of gestation does not significantly impact their predictive accuracy. These findings support the integration of Doppler ultrasound into clinical practice for the early identification of PE risk.

Ultrasound allows for the assessment of placental thickness, echotexture, and vascularization [108]. A small, abnormally shaped, or heterogeneous placenta may indicate placental insufficiency, which is a key pathophysiological factor in PE development [109].

Doppler evaluation of placental circulation, including the umbilical artery and fetal middle cerebral artery, provides further insight into placental function and fetal adaptation to hypoxia [110]. Several studies have highlighted the role of Doppler ultrasound in assessing hemodynamic changes and predicting adverse pregnancy outcomes in preeclamptic pregnancies. Rose et al. found significant correlations between umbilical artery (UA) and middle cerebral artery (MCA) Doppler indices, with stronger associations in normotensive pregnancies compared to preeclamptic cases [111]. Zhao et al. reported that high-risk pregnancies exhibited increased UA indices and decreased MCA indices, emphasizing their value in predicting fetal complications [112]. Tasci et al. observed significantly higher UA Doppler indices and lower MCA indices in preeclamptic pregnancies, particularly in cases with intrauterine FGR, reinforcing the importance of combined Doppler assessments in improving diagnostic accuracy [112]. Zhou et al. demonstrated that severe pre-eclampsia was associated with increased UA and uterine artery (UtA) indices and decreased MCA indices, with color Doppler ultrasound effectively predicting adverse pregnancy outcomes [110].

PE is often associated with intrauterine FGR [113]. Serial fetal biometry, including head circumference, abdominal circumference, femur length, and estimated fetal weight, helps detect growth abnormalities. Oligohydramnios, or reduced amniotic fluid volume, can be an indirect indicator of uteroplacental insufficiency and pre-eclampsia-related complications [114,115]. Ultrasound-based biophysical profile scoring, which includes fetal movements, tone, breathing, and amniotic fluid assessment, contributes to the overall evaluation of fetal well-being in pregnancies complicated by hypertensive disorders.

### 5.4. Combined and Machine Learning-Based Models for Risk Prediction

Identifying women at high risk of developing PE later in pregnancy is a key objective of first-trimester screening, enabling the timely introduction of effective preventive strategies. Currently, many centers do not use a combined first-trimester screening method, and high-risk women are often identified solely through the evaluation of clinical risk factors, as outlined in the ACOG and NICE guidelines [116,117]. Integrating clinical risk factors, maternal blood pressure (including mean arterial pressure (MAP), mean UtA PI, and maternal angiogenic biomarkers into a unified algorithm could offer a more precise method for identifying women at high risk of developing PE [116,118].

Combination models include first-trimester screening, which combines maternal history, blood pressure, biochemical markers, and uterine artery Doppler assessments to stratify risk early in pregnancy. Second- and third-trimester monitoring uses serial assessments of biochemical markers and Doppler studies to refine risk estimation and guide clinical management [116]. Emerging technologies leverage large datasets to improve predictive modeling for PE, potentially revolutionizing early detection and intervention strategies.

Conventional approaches to risk prediction mainly focus on detecting risk factors and applying classical statistical models, such as multiple logistic regression and Bayesian principles [119,120]. However, these approaches often require complex formulas, variable prediction indicators, and lack external validation, limiting their clinical application. In response, machine learning (ML) algorithms have emerged as promising tools for improving prediction accuracy.

A systematic review comparing 16 ML algorithms with 84 classical regression models found that ML approaches generally outperformed traditional methods in predicting PE [121]. In studies that evaluated both methods, ML models demonstrated superior predictive performance in eight out of ten cases. Frequently used prognostic indicators included maternal demographic and clinical characteristics, along with biochemical markers such as PAPP-A and PlGF, as well as biophysical markers like UtA-PI and MAP.

The most effective ML algorithms were random forest, gradient boosting, and extreme gradient boosting. For instance, an elastic net algorithm incorporating maternal characteristics and routine prenatal laboratory data achieved an area under the curve (AUC) of 0.79 for PE prediction and 0.89 for early-onset PE [122]. Similarly, a study using stochastic gradient boosting reported the highest accuracy (0.973) and the lowest false-positive rate (0.009) when predicting late-onset PE [123]. Another study conducted by Li et al. using extreme gradient boosting with 38 clinical parameters achieved an AUC of 0.955, identifying fasting plasma glucose, mean blood pressure, and body mass index as the most predictive features [124].

Despite their promise, most ML models lack external validation and deployment strategies. These models provide benefits by processing raw biomarker data without requiring conversion to multiples of the median (MoMs) and by incorporating a wide range of prognostic factors to enhance predictive accuracy. Continued refinement and validation of ML models are crucial for their broader clinical application in PE risk prediction.

To translate the predictive power of ML models into real-world clinical benefit, it is essential to establish practical implementation pathways within routine obstetric care. One promising avenue is the integration of ML algorithms into existing electronic health record (EHR) systems. Embedding ML models directly into EHR platforms can facilitate automated risk calculation in real-time, utilizing routinely collected antenatal data such as maternal demographics, medical history, blood pressure measurements, laboratory results, and ultrasound findings.

This approach has demonstrated strong potential in multiple studies. For instance, a study of 3759 pregnancies from Xinhua Hospital (Shanghai Jiaotong University) applied several ML models—including XGBoost—based on 38 clinical features routinely collected in the early second trimester. The XGBoost model showed excellent predictive performance (accuracy = 0.920, auROC = 0.955), identifying fasting plasma glucose, mean blood pressure, and BMI as key predictors. Even a simplified version of the model, using only self-reported features, achieved an auROC of 0.83 [124].

In a large multicenter study analyzing 108,557 pregnancies across the Mount Sinai Health System, researchers developed a digital phenotyping pipeline and ML models to predict pre-eclampsia at different stages of pregnancy. The models achieved AUCs of 0.92 (antepartum), 0.82 (intrapartum), and 0.89 (postpartum), and identified both known and novel predictors, such as CBC-related markers, providing a foundation for precision risk assessment [125].

Another retrospective cohort study from Lucile Packard Children’s Hospital at Stanford, using data from over 16,000 births, demonstrated the power of statistical learning models—including elastic net and gradient boosting—to predict both overall and early-onset pre-eclampsia using routine data collected before 16 weeks’ gestation. The elastic net model achieved an AUC of 0.79 for any PE and 0.89 for early-onset PE, with a high true-positive rate (72.3%) and a low false-positive rate (8.8%) for early-onset cases, emphasizing the feasibility of early identification through routine prenatal care [126].

This seamless integration of predictive tools into clinical workflows would enable clinicians to receive instant alerts when a patient is identified as high-risk for PE, prompting timely interventions such as the initiation of low-dose aspirin, enhanced monitoring, or referral to a specialist. Furthermore, EHR-based decision support systems could generate personalized risk reports, aiding shared decision making between providers and patients.

To support widespread adoption, ML models must be user-friendly, interpretable, and compatible with various clinical workflows [127]. Developing clinician-facing dashboards that visualize risk trajectories over time, explain contributing factors, and provide evidence-based management options could improve usability and trust. Integration with national or regional maternal health databases would also support continuous model refinement and benchmarking.

Additionally, incorporating ML tools into telemedicine platforms could expand access in low-resource or rural settings, where specialist care is limited. Cloud-based solutions may allow for remote risk assessment and centralized expert review, increasing equity in prenatal care.

Ultimately, the success of ML-enhanced screening for PE will depend on close collaboration between clinicians, data scientists, health informaticians, and policymakers. Clear regulatory pathways, rigorous external validation, and prospective impact studies will be essential to ensure these models are safe, effective, and ethically deployed at scale.

However, the implementation of ML-based predictive models in low-income countries (LICs) presents significant challenges that must be addressed to ensure equitable maternal healthcare [128]. In many LICs, access to basic prenatal services, diagnostic equipment, and reliable laboratory infrastructure remain limited [129,130]. Routine data required for ML models—such as blood pressure measurements, laboratory markers, or even consistent gestational age dating—may be inconsistently collected or entirely unavailable. Furthermore, EHR systems are often underdeveloped or non-existent, making real-time data integration and automated risk calculation difficult [131].

The digital divide poses another barrier, as limited internet access, lack of technical support, and inadequate training of healthcare personnel can hinder the deployment and maintenance of ML tools [132]. Additionally, ML models developed in high-income countries (HICs) may not generalize well to LIC populations due to differences in genetics, environmental exposures, healthcare access, and comorbidities [133,134]. This underscores the urgent need for locally validated models that are built using data that reflect the realities of maternal health in resource-limited settings.

To bridge these gaps, global health initiatives must prioritize infrastructure development, workforce training, and the creation of open-access, low-resource-adapted ML tools. Cloud-based or mobile applications that can operate offline or with minimal input data offer one possible solution. Collaborative efforts involving local healthcare providers, governments, and international organizations are essential to develop, validate, and implement context-specific models that are both accurate and feasible in LIC settings.

## 6. Current Strategies for the Prevention of Pre-Eclampsia

Research has confirmed that administering low-dose aspirin significantly decreases the likelihood of developing PE and mitigates its associated adverse outcomes, such as preterm birth and FGR, by approximately 10 to 20 percent in patients at moderate to high risk. With a strong maternal and fetal safety profile, it is considered a reasonable preventive approach for these individuals. The rationale behind its use stems from observations that PE is linked to increased platelet turnover and elevated levels of platelet-derived thromboxane [135,136,137]. Extensive clinical trials have investigated the effectiveness of low-dose aspirin in reducing PE risk among high-risk individuals. Unlike higher doses, low-dose aspirin (typically 60–150 mg/day) selectively inhibits platelet thromboxane synthesis while preserving endothelial prostacyclin production, thereby improving placental blood flow and reducing the likelihood of PE-related complications [137,138]. Since thromboxane promotes platelet aggregation and vasoconstriction, whereas prostacyclin has the opposite effect, this mechanism likely contributes to aspirin’s protective role. Additionally, although not extensively studied, its benefits may also be related to its modulation of the exaggerated inflammatory response observed in pre-eclampsia.

Meta-analyses of randomized trials have consistently demonstrated the efficacy of aspirin in reducing the risk of PE and its complications [139]. A 2019 meta-analysis of 74 trials involving over 40,000 patients, spanning different risk levels, found that low-dose aspirin (50 to 162 mg/day) significantly reduced proteinuric PE (16 fewer cases per 1000 patients, RR 0.82, 95% CI 0.77–0.88), fetal or neonatal mortality (5 fewer deaths per 1000, RR 0.85, 95% CI 0.76–0.95), preterm birth before 37 weeks (16 fewer cases per 1000, RR 0.91, 95% CI 0.87–0.95), and small-for-gestational-age newborns (7 fewer cases per 1000, RR 0.84, 95% CI 0.76–0.92) [140]. The composite of serious maternal and neonatal adverse outcomes was also lower (20 fewer cases per 1000, RR 0.90, 95% CI 0.85–0.96). While a minor elevation in the risk of postpartum hemorrhage exceeding 500 mL was noted (RR 1.06, 95% CI 1.00–1.12), no statistically significant association was found with placental abruption (RR 1.21, 95% CI 0.95–1.54). Earlier research indicated that aspirin could lower the risk of preterm birth by nearly 60% before 32 weeks (1.2% vs. 2.9%, OR 0.42, 95% CI 0.19–0.93). However, the findings of this meta-analysis demonstrated only a slight reduction (RR 0.92, 95% CI 0.83–1.02). Furthermore, aspirin did not significantly impact the risk of HELLP syndrome (RR 0.77, 95% CI 0.44–1.36), severe maternal morbidity (RR 1.00, 95% CI 0.72–1.39), or neonatal special care unit admission (RR 0.95, 95% CI 0.90–1.00) [140].

Another meta-analysis conducted by the USPSTF in 2021 focused on 23 trials involving nearly 27,000 patients, most of whom were at increased risk of PE based on clinical risk factors. This study found that aspirin reduced the incidence of PE (absolute risk reduction [ARD] −4.1 percent, 95% CI −8.4 to −1.3; RR 0.85, 95% CI 0.75–0.95), perinatal mortality (ARD 0.0 percent, 95% CI −1.1 to 0.5; RR 0.79, 95% CI 0.66–0.96), preterm birth before 37 weeks (ARD −5.7 percent, 95% CI −12.9 to −3.0; RR 0.80, 95% CI 0.67–0.95), and FGR or small-for-gestational-age newborns (ARD −4.6 percent, 95% CI −8.9 to −0.2; RR 0.82, 95% CI 0.68–0.99). Importantly, no significant increase in bleeding-related complications was observed in this analysis [141].

A meta-analysis conducted in 2018 focused on both preterm and term PE, revealing that aspirin notably decreased the likelihood of preterm PE occurring before 37 weeks (RR 0.62, 95% CI 0.45–0.87). However, its impact on term PE was not statistically significant (RR 0.92, 95% CI 0.70–1.21) [142]. One of the key trials included in this analysis, the ASPRE trial, identified high-risk individuals using a multivariable first-trimester screening algorithm and randomly assigned them to receive 150 mg of aspirin daily or placebo from 11 to 13 weeks of gestation until 36 weeks. The aspirin group showed a 62 percent reduction in preterm PE before 37 weeks (1.6 vs. 4.3 percent, OR 0.38, 95% CI 0.20–0.74) and a possible even greater reduction before 34 weeks (0.4 vs. 1.8 percent, OR 0.18, 95% CI 0.03–1.03). However, the reduction in term PE was not significant (6.6 vs. 7.2 percent, OR 0.95, 95% CI 0.57–1.57) [143].

While the use of low-dose aspirin is generally considered safe and effective for the prevention of PE, certain contraindications and population-specific considerations warrant careful attention. Contraindications include known hypersensitivity to aspirin, active peptic ulcer disease, bleeding disorders, or a history of gastrointestinal or intracranial hemorrhage [141]. Although most meta-analyses report minimal increases in bleeding risk, a slight but statistically significant rise in postpartum hemorrhage has been observed in some studies, especially when aspirin is continued late into pregnancy [144]. Therefore, the timing of discontinuation—typically recommended by 36 weeks’ gestation—is crucial to mitigate these risks [145]. Additionally, population-specific factors such as maternal comorbidities, ethnicity, and access to antenatal care may influence aspirin’s efficacy. For instance, emerging evidence suggests that aspirin may be less effective in populations with higher baseline risks, such as those with chronic hypertension or obesity, and optimal dosing and timing might differ in these groups [146]. Shared decision making, informed by individualized risk assessment and clinical judgment, remains essential when initiating aspirin prophylaxis for PE prevention.

Insufficient dietary calcium intake has been associated with an increased risk of hypertension [143]. To meet daily requirements, pregnant individuals are advised to obtain adequate calcium either through their diet or supplementation—1000 mg per day for those aged 19–50 and 1300 mg per day for adolescents [147]. Since the average intake among reproductive-aged females is about 950 mg/day, most may require modest supplementation. Those with low dairy intake or those living in regions with calcium deficiency may benefit from higher doses to reduce the risk of PE [148].

For individuals with low dietary calcium intake, especially those at risk of hypertension, the World Health Organization (WHO) advises a daily calcium intake of 1500–2000 mg [149]. A 2022 meta-analysis of over 20,000 participants found that calcium supplementation significantly reduced PE risk, especially in individuals with low baseline calcium intake. Both low- and high-dose supplementation (≥1 g/day) were effective [150]. Another large trial comparing 500 mg to 1500 mg supplementation found that the lower dose was noninferior, suggesting a single 500 mg supplement may be a more practical alternative to the WHO’s higher-dose recommendation [151].

Pre-pregnancy weight loss and appropriate gestational weight gain also lower the risk of PE, particularly for overweight or obese individuals. Studies show that weight loss before pregnancy and avoiding excessive weight gain during pregnancy reduce PE risk [152,153].

Exercise may also help prevent PE, especially for those not eligible for low-dose aspirin prophylaxis. A meta-analysis found that exercising at least three times a week for 25 min per session lowered the PE risk [154]. Supervised exercise programs, including aerobic and strength training, were particularly effective in reducing hypertensive disorders in pregnancy [155].

## 7. Innovations and Future Directions

Advancements in artificial intelligence (AI) and ML are revolutionizing the early detection and prevention of PE by integrating vast amounts of clinical, biochemical, and imaging data to develop predictive models with high sensitivity and specificity [156]. Future research in this field should focus on several key areas to enhance screening efficacy and clinical applicability.

A key future advancement lies in developing multimodal AI-based screening systems that integrate multiple data sources, including maternal demographics, clinical history, biochemical markers, and advanced imaging methods [157]. By integrating these diverse datasets, AI-driven algorithms can provide more accurate risk stratification and enable early interventions.

One of the critical challenges in adopting AI-based screening tools is the lack of transparency in how predictions are made [158]. Future research should emphasize explainable AI (XAI) approaches to ensure that ML models provide interpretable outputs for clinicians. This will enhance trust, facilitate clinical decision making, and promote integration into routine prenatal care.

To improve model generalizability and reduce biases associated with regional or ethnic variations, federated learning techniques should be explored. This approach allows multiple healthcare institutions to collaboratively train AI models on decentralized datasets while maintaining data privacy. Establishing global AI consortia for PE prediction could significantly enhance model robustness and applicability across diverse populations.

The proliferation of wearable devices and mobile health applications presents a unique opportunity to enhance PE screening [159,160]. Future AI-driven models could incorporate real-time physiological data, such as blood pressure variability, heart rate, and sleep patterns, to improve early detection and risk prediction. Mobile applications equipped with AI-powered decision support systems could facilitate home-based monitoring and timely medical interventions.

AI and ML models hold the potential to move beyond population-based risk assessments toward personalized screening strategies. Future research should focus on developing individualized risk prediction models that account for genetic predisposition, lifestyle factors, and personalized treatment responses. By tailoring preventive measures, such as aspirin prophylaxis or dietary modifications, based on AI-generated risk profiles, clinicians can optimize maternal and fetal outcomes.

Despite promising advancements, AI-driven screening models require rigorous prospective validation in large-scale clinical trials before widespread adoption. Future research should prioritize validating model performance in real-world clinical settings, ensuring adherence to regulatory frameworks, and obtaining necessary approvals for clinical implementation.

## 8. Conclusions

PE remains a major obstetric challenge with significant maternal and fetal complications. Advances in understanding its pathophysiology have led to the identification of key risk factors and the development of various screening methods, ranging from clinical risk assessment to biochemical markers, ultrasound parameters, and machine-learning-based models. While early screening is crucial for timely intervention, current preventive strategies show varying degrees of effectiveness.

Emerging innovations, such as novel biomarkers, artificial intelligence-driven predictive models, and potential therapeutic agents, offer promising avenues for improving early detection and prevention. However, further research is needed to refine these strategies and enhance their clinical applicability. A multidisciplinary approach integrating modern screening tools with personalized prevention plans could significantly reduce the burden of pre-eclampsia and improve pregnancy outcomes.

## Figures and Tables

**Table 1:** Maternal risk factors for pre-eclampsia identified by professional organizations [60].

Professional Organization	High-Risk Factors	Moderate Risk Factors	Indication for Aspirin Use
NICE, 2019 (United Kingdom), updated in 2023	Previous pregnancy with PE Chronic hypertension Autoimmune disease T1DM/T2DM Chronic kidney disease Antiphospholipid syndrome	Nulliparity Age, ≥40 y Interpregnancy interval, >10 y BMI at first visit, ≥35 kg/m^2^ Family history of PE Multifetal pregnancy	1 or more high-risk factors 2 or more moderate-risk factors Dose: 75 to 150 mg/d from 12 weeks until delivery
ACOG, 2018 (USA), updated in 2020	Previous pregnancy with PE Chronic hypertension Autoimmune disease (systemic lupus erythematosus, the antiphospholipid syndrome) T1DM/T2DM Renal disease Multifetal gestation	Nulliparity Age, ≥35 y Interpregnancy interval, >10 y Obesity (BMI, >30 kg/m^2^) Family history of PE (mother or sister) History of SGA or adverse outcome Sociodemographic characteristics (African American race or low socioeconomic status)	1 or more high-risk factors 2 or more moderate-risk factors Dose: 81 mg/d initiated between 12 and 28 weeks (better before 16 weeks), until delivery
ISSHP, 2018	Prior PE Chronic hypertension Pregestational diabetes mellitus BMI, >30 kg/m^2^ Chronic kidney disease Antiphospholipid syndrome	Advanced maternal age, >35 y Family history of PE Short duration of sexual relationship (<6 mo) before the pregnancy Primiparity Primipaternity Connective tissue disorder	1 or more high-risk factors 2 or more moderate risk factors Dose: 100 to 150 mg/d start before 16 weeks until 37 weeks
SOGC, 2014, updated in 2022	Previous 3y Chronic hypertension Renal disease T1DM/T2DM Autoimmune disease (systemic lupus erythematosus, antiphospholipid syndrome) Chronic vascular disease Multifetal gestation Obesity (BMI ≥ 30 kg/m^2^)	Age ≥ 40 years Nulliparity Family history of PE (mother or sister) Interval of more than 10 years since the last pregnancy First-trimester BMI ≥ 25 kg/m^2^ Interpregnancy interval ≤ 2 years History of preterm birth IVF pregnancy	1 or more high-risk factors 2 or more moderate risk factors Dose: 81 to 162 mg/d start from before 16 weeks until delivery

Abbreviations: PE—pre-eclampsia, NICE—National Institute for Health and Care Excellence, ACOG—American College of Obstetricians and Gynecologists, ISSHP—International Society for the Study of Hypertension in Pregnancy, SOGC—Society of Obstetricians and Gynaecologists of Canada, T1DM—Type 1 diabetes mellitus, T2DM—Type 2 diabetes mellitus, BMI—body mass index, USA—United States of America, SGA—small for gestational age, IVF—in vitro fertilization.

**Table 2:** Economic impact of sFlt-1/PlGF ratio testing in different countries.

Country	Study	Key Findings	Estimated Cost Savings per Patient
United States	Khosla et al., 2021 [94]	Implementation of the sFlt-1/PlGF ratio test reduced hospital admissions by 34–49%	USD 1050
Germany	Schlembach et al., 2018 [95]	Use of the sFlt-1/PlGF ratio test decreased hospitalizations from 44.6% to 24.0%	EUR361
Netherlands	Wind et al., 2022 [96]	Combining sFlt-1/PlGF ratio testing with telemonitoring reduced hospital admissions by 41% and outpatient visits by 36%	EUR46
Italy	Frusca et al., 2017 [97]	Introduction of the sFlt-1/PlGF ratio test reduced management costs from EUR 2384 to EUR 1714 per patient	EUR 670
Switzerland	Hodel et al., 2019 [98]	The test helped stratify patients, potentially reducing unnecessary hospitalizations and associated costs.	EUR 345
United Kingdom	Vatish et al., 2016 [99]	The economic analysis suggests that introduction of the test could reduce the number of women hospitalized by more than half (56%), from 36% to 16%	GBP 344
Japan	Ohkuchi et al., 2021 [100]	Introduction of the sFlt-1/PlGF ratio test using a cutoff value of 38 resulted in a reduced hospitalization rate compared with the rate in the no-test scenario (14.4% versus 8.7%)	JPY 16 373
Argentina	Garay et al., 2022 [101]	Nationwide implementation of the sFlt-1/PlGF test could save approximately ARS 6987 million annually, reducing costs by 39.1% through better patient triage.	ARS 80 504
Brazil	Figueira et al., 2018 [102]	The sFlt-1/PlGF test reduced unnecessary hospitalizations and resulted in cost savings in both settings: public hospitals and private hospitals.	BRL 185.06 (public hospital) BRL 635.84 (private hospital)
Colombia	Duva et al., 2017 [103]	In Colombia, a five-year budget impact analysis projected that implementing the sFlt-1/PlGF test could save the public healthcare system 47 billion Colombian pesos. This equates to significant cost savings, primarily driven by a reduction in hospitalizations—from 36% in the standard care scenario to 16% with the use of the sFlt-1/PlGF test.	COP 182 841
China	Chen et al., 2019 [104]	The pre-eclampsia cost ‘no-test’ group about EUR 1482 per patient and it cost ‘test’ group EUR 1134 per patient.	EUR 348
